# Identification of microenvironment related potential biomarkers of biochemical recurrence at 3 years after prostatectomy in prostate adenocarcinoma

**DOI:** 10.18632/aging.203121

**Published:** 2021-06-16

**Authors:** Xiaoru Sun, Lu Wang, Hongkai Li, Chuandi Jin, Yuanyuan Yu, Lei Hou, Xinhui Liu, Yifan Yu, Ran Yan, Fuzhong Xue

**Affiliations:** 1 Department of Biostatistics, School of Public Health, Cheeloo College of Medicine, Shandong University, Jinan 250012, Shandong, China; 2 Institute for Medical Dataology, Cheeloo College of Medicine, Shandong University, Jinan 250012, Shandong, China

**Keywords:** prostate adenocarcinoma, biochemical recurrence, gene expression, tumor microenvironment, targeted maximum likelihood estimation

## Abstract

Prostate adenocarcinoma is one of the leading adult malignancies. Identification of multiple causative biomarkers is necessary and helpful for determining the occurrence and prognosis of prostate adenocarcinoma. We aimed to identify the potential prognostic genes in the prostate adenocarcinoma microenvironment and to estimate the causal effects simultaneously. We obtained the gene expression data of prostate adenocarcinoma from TCGA project and identified the differentially expressed genes based on immune-stromal components. Among these genes, 68 were associated with biochemical recurrence at 3 years after prostatectomy in prostate adenocarcinoma. After adjusting for the minimal sets of confounding covariates, 14 genes (*TNFRSF4, ZAP70, ERMN, CXCL5, SPINK6, SLC6A18, CHRM2, TG, CLLU1OS, POSTN, CTSG, NETO1, CEACAM7*, and *IGLV3-22*) related to the microenvironment were identified as prognostic biomarkers using the targeted maximum likelihood estimation. Both the average and individual causal effects were obtained to measure the magnitude of the effect. CIBERSORT and gene set enrichment analyses showed that these prognostic genes were mainly associated with immune responses. *POSTN* and *NETO1* were correlated with androgen receptor expression, a main driver of prostate adenocarcinoma progression. Finally, five genes were validated in another prostate adenocarcinoma cohort (GEO: GSE70770). These findings might lead to the improved prognosis of prostate adenocarcinoma.

## INTRODUCTION

Prostate cancer is a common malignant tumor and the leading cause of cancer-related mortality in men [1]. Prostate adenocarcinoma (PRAD) is the most common type of prostate cancer, whereas other types of prostate cancer are relatively rare [2]. The duration between surgery and prostate-specific antigen-defined biochemical recurrence (BCR) (≤ 3 vs. > 3 years) after definitive local therapy is a significant risk factor for defining specific mortality of prostate cancer [3]. Approximately 35% of men who undergo radical prostatectomy have been reported to experience BCR within 10 years [3, 4]. Hence, exploration of new mechanisms of BCR using integrated bioinformatics analysis could be applied to stratify patients at risk and guide the decision-making for treatment.

Many studies have shown that the tumor microenvironment (TME) is implicated in the development and sustained growth, invasion, and metastasis of cancer [5–8]. Infiltrating immune and stromal cells are important components of the TME and have been shown to significantly influence the progression of malignancy [5, 6]. Evidence has suggested that interactions between tumor cells and stroma mediate the development of cancer and tissue preferences for metastasis [9]. In PRAD, the stromal cells express the androgen receptor (AR), which is the main driver of prostate cancer pathogenesis and progression [10, 11]. However, it is still a challenging undertaking to explore the causal effects of gene expressions on the prognosis of patients with PRAD in the TME. In this study, we focused on exploring the prognostic genes in the TME based on the immune and stromal scores, and then detected their causal effects on the PRAD BCR.

Using observational data such as the online databases, the Cancer Genome Atlas (TCGA), to detect causative biomarkers and estimate causal effects is difficult because of the unbalanced distribution of pretreatment variables between treatment groups, henceforth covariates [12, 13]. Several methods have been proposed to overcome these problems, including previously applied propensity scores, inverse probability weighting, and g-computation [14, 15]. These methods rely on the consistent estimations of the exposure or outcome mechanism. In this study, we used targeted maximum likelihood estimation (TMLE), a doubly robust method to detect the prognostic genes and estimate both the average and individual causal effects [16–18]. TMLE is a semi-parametric method that flexibly establishes the causal models using multiple machine learning methods, which requires weaker assumptions than other common models.

When estimating the causal effect of the prognostic gene on the BCR status, controlling too many covariates might result in the poor performance of the estimator [19–21]. Luna et al. proposed the algorithm *CovSel* for covariate selection, which reduced the dimension of the covariate set for estimation of the causal effect [20, 22]. Furthermore, Loh et al. compared *CovSel* with other covariate selection methods, such as collaborative-TMLE and augmented backward elimination, to evaluate their ability to correctly select confounders and control the type I error rate after data-driven covariate selection. They found that *CovSel* selected at least one confounder each time and had an approximate 70% probability to select the sufficient confounders exactly. Additionally, *CovSel* approximately controlled the type I error empirically at the significance level [23]. Thus, we applied the algorithm *CovSel* to select the minimal conditioning set that was sufficient for unbiased effect estimations of the target gene on the BCR.

**Figure 1:**
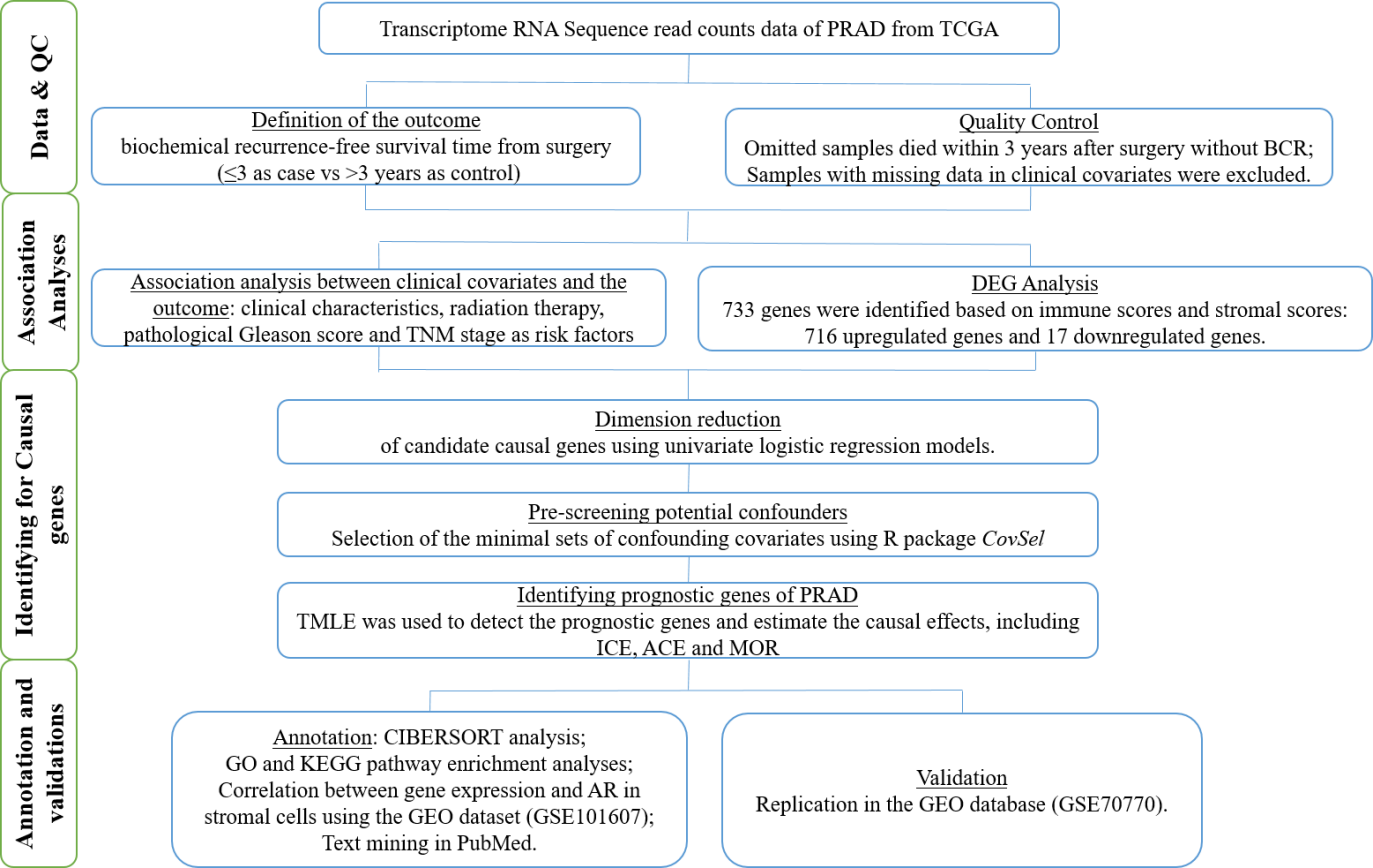
The workflow schematic for identifying prognostic biomarkers in PRAD.

We accordingly obtained an adult PRAD patient dataset from TCGA project to identify the potential prognostic genes that were related to the TME and caused BCR at 3 years after prostatectomy. Moreover, the causal effects of these genes were estimated for individual therapies. We verified these prognostic genes using the Gene Expression Omnibus (GEO) database. We drew a workflow schematic to illustrate the entire study design for the identification of the prognostic biomarkers in PRAD (Figure 1).

## RESULTS

### Clinical and pathological characteristics of men with PRAD from TCGA

We obtained the gene expression profiles and clinical information of the PRAD patients with an initial pathologic diagnosis made between 2000 and 2013 from TCGA. A total of 209 patients were included after the exclusion of the subjects according to the exclusion criteria in Methods. Among them, 112 patients (53.6%) had BCR within 3 years after definitive local therapy, whereas 97 patients (46.4%) had no BCR within 3 years (Table 1). Based on the Estimation of STromal and Immune cells in MAlignant Tumour tissues using Expression data (ESTIMATE) algorithm [24], the stromal scores were obtained and distributed between -1,867.0 and 1,789.3, and the immune scores ranged from -1,796.65 to 2962.96. Patients with BCR within 3 years had lower immune and ESTIMATE scores than patients without BCR (*P* < 0.05). We identified radiation therapy, pathological Gleason score and tumor-node-metastasis (TNM) stage as significant risk factors for BCR (*P* < 0.05), which were selected as candidate covariates.

**Table 1:** Clinical and pathological characteristics of 209 PARD patients from TCGA.

**Clinic pathologic variable**	**BCR (n=112)**	**BCR-free (n=97)**	**Total (n=209)**	**P value**
**Age (years)**	60.74 (6.84)	61.51 (6.09)	61.1 (6.50)	0.397
**Stromal scores**	-533.82 (459.27)	-402.32 (607.96)	-472.79 (536.16)	0.076
**Immune scores**	-471.57 (587.37)	-263.78 (789.91)	-375.14 (694.90)	0.03
**ESTIMATE scores**	-1,005.4 (930.04)	-666.11 (1,296.03)	-847.93 (1,124.99)	0.028
**Weight**	197.5 (181.27)	316.25 (430.36)	267.11 (353.25)	0.072
**Radiation therapy**				
Yes	11 (9.82)	22 (22.68)	33 (15.79)	0.019
No	101 (90.18)	75 (77.32)	176 (84.21)	
**Gleason score**				
6	6 (5.36)	1 (1.03)	7 (3.35)	< 0.001
7	67 (59.82)	25 (25.77)	92 (44.02)	
8~9	39 (34.82)	71 (73.20)	110 (52.63)	
**TNM stage**				
I	5 (4.46)	1 (1.03)	6 (2.87)	< 0.001
II	37 (33.04)	9 (9.28)	46 (22.01)	
III	54 (48.21)	59 (60.82)	113 (54.07)	
IV	16 (14.29)	28 (28.87)	44 (21.05)	

*Data are mean ± SD and frequency (percent) for numeric and category variables, respectively.

### Comparison of gene expression profiles in PRAD according to the immune and stromal scores

**Figure 2:**
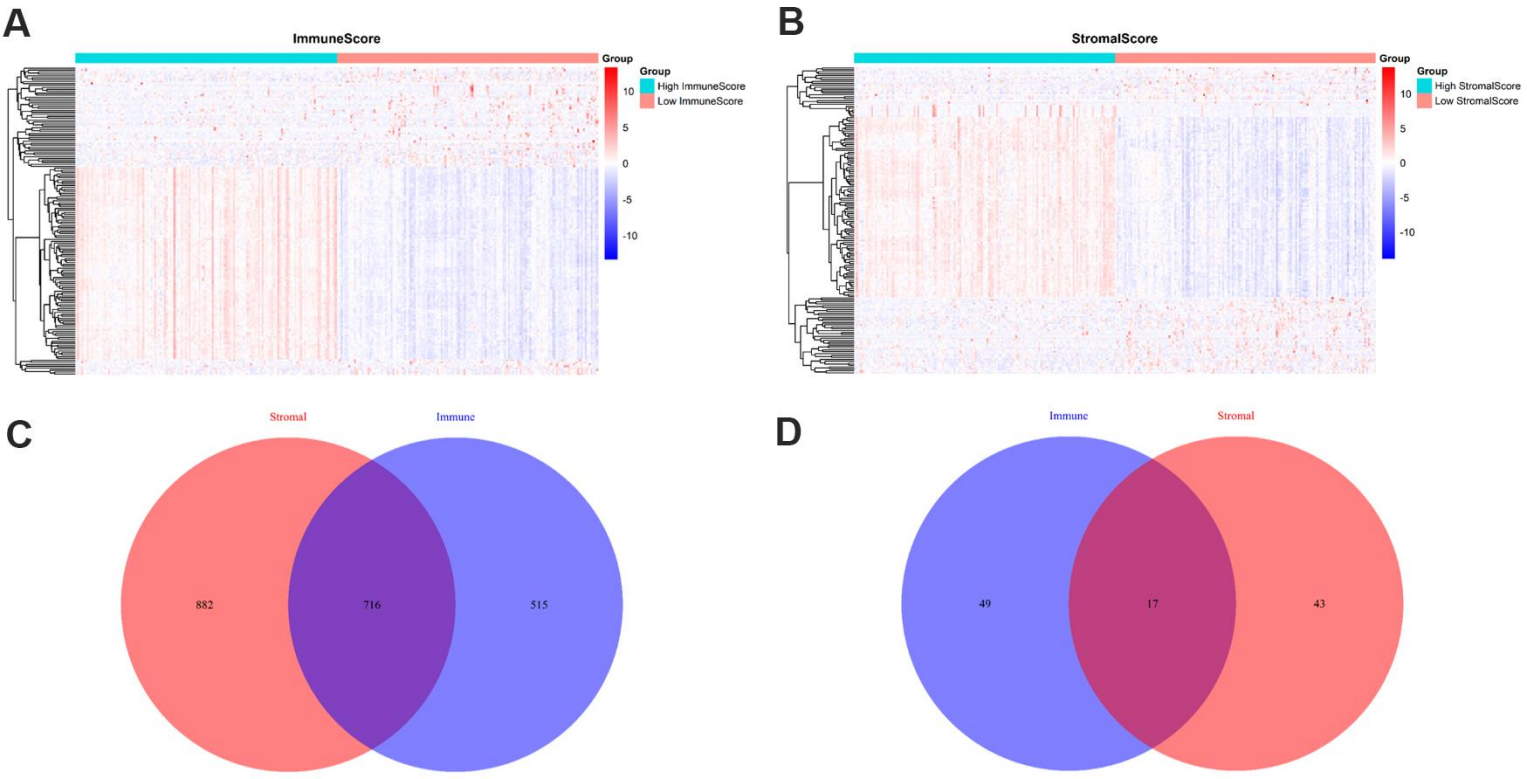
**Comparison of gene expression profiles with immune and stromal scores.** (**A**) A heat map of DEGs between the high and low immune score groups; (**B**) A heat map of DEGs between the high and low stromal score groups; (**C**) Venn diagrams showing the number of high expression and (**D**) low expression of DEGs in both immune and stromal score groups.

To identify the differentially expressed genes (DEGs), we divided the 209 PRAD patients to high and low score groups according to their immune and stromal scores. A total of 112 patients (53.59%) had high stromal scores, and 115 (55.02%) had high immune scores. Based on immune scores, 515 gene expression levels were demonstrated to be increased and 49 genes were decreased in the high score group as compared to the low score group (Figure 2A). Similarly, for the high and low groups based on stromal scores, 882 genes were increased and 43 genes were decreased (Figure 2B). log2|fold change| > 1 and False discovery rate (FDR) < 0.05 were used as the criteria for screening DEGs. Moreover, the Venn diagrams (Figure 2C, 2D) showed that 716 genes had high expressions in both high immune and stromal score groups, and 17 genes had low expressions. These overlapped DEGs (733 genes in total) might be the determinants of TME status. Thus, we decided to focus on these DEGs for all subsequent analyses.

### Correlation between the expression of differentially expressed genes and biochemical recurrence

**Figure 3:**
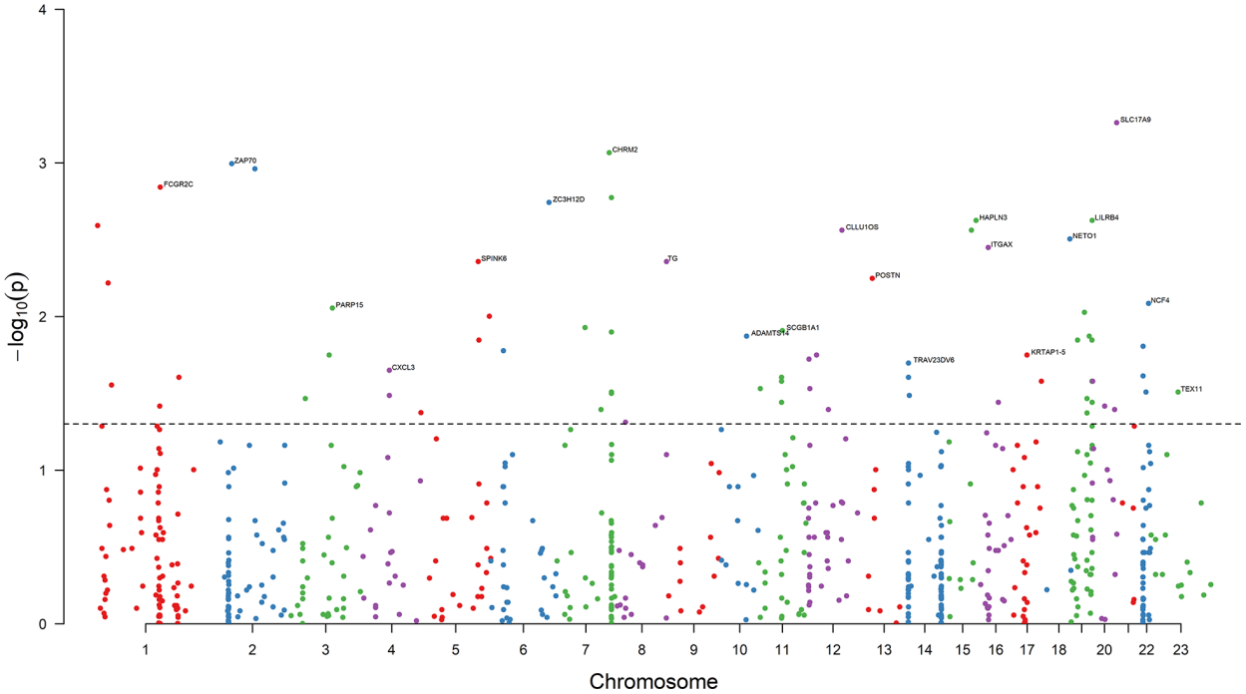
**Results of association analysis.** The dashed black line is the bound of *P* = 0.05. 68 DEGs are associated with the PRAD BCR (*P* < 0.05). The top genes with the minimum *P* value on each chromosome are annotated. Chromosome 23 is denoted Chromosome X.

Results of the univariate logistic regression models showed that 68 DEGs were associated with BCR in PRAD (*P* < 0.05), and thus we selected them as candidate causative genes involved in immune and stromal cells (Figure 3).

### Selection of confounding covariates for the minimal sets of confounding covariates

**Figure 4:**
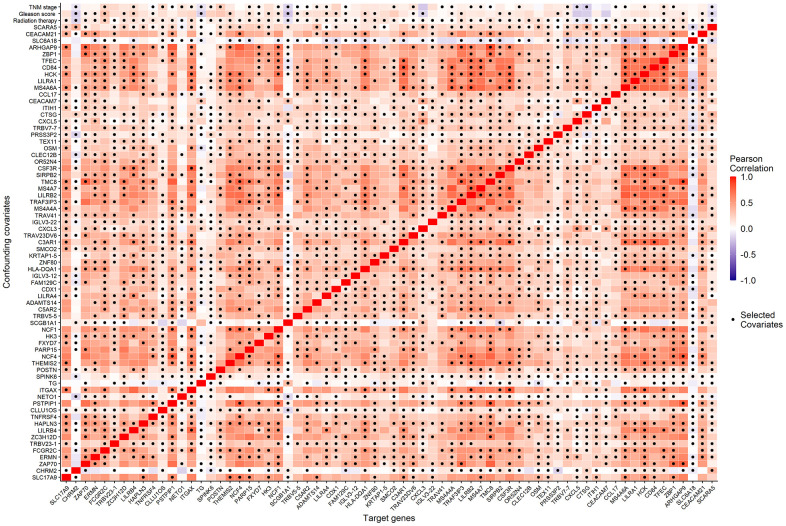
The Pearson correlation coefficients (the corresponding color) of the 68 candidate genes (horizontal axis) and the 70 candidate confounding covariates (vertical axis), as well as the minimal confounding covariate set of each candidate gene (the dark dot of each column).

We considered the 68 associated genes along with 3 significant clinical covariates (radiation therapy, pathological Gleason score, and TNM stage) as the candidate confounding covariates. To reduce the dimension of confounding covariates for improved causal effect estimations, we selected the minimal sets of confounders between each candidate causative gene and BCR status using the R package, *CovSel*. *W_G_* is a subset of the candidate confounding covariates that leads to other covariates after removing *W_G_* conditionally independent of the outcome *Y* given *W_G_*. *W_G_* is illustrated in Supplementary material (Supplementary Figure 1). *V_G_* is a subset of *W_G_* after removing variables conditionally independent of the target gene *G* given *V_G_*. Figure 4 shows the Pearson correlation coefficients (displayed in color from dark blue to red as correlations from -1 to 1) of the 68 candidate genes and the corresponding 70 candidate confounding covariates of each gene, as well as the selected confounding covariates *V_G_* for each candidate gene (the dark dot of each column).

### Selection of prognostic genes and causal effect estimations using targeted maximum likelihood estimation

To explore the prognostic genes of BCR, we estimated the causal effects (the average causal effect (ACE), the marginal odds ratio (MOR), and the individual causal effect (ICE)) of the 68 candidate genes on early-onset BCR using TMLE. Considering the complex network and interactions among these genes, we collaborated the Super Learner with TMLE and obtained a weighted causal effect of these models. Accordingly, we identified 14 prognostic genes (*TNFRSF4*, *ZAP70*, *ERMN*, *CXCL5*, *SPINK6*, *SLC6A18*, *CHRM2*, *TG*, *CLLU1OS*, *POSTN*, *CTSG*, *NETO1*, *CEACAM7*, and *IGLV3-22*) on 12 chromosomes with *P*-ACE < 0.05 (Table 2). 7 out of these genes (*ZAP70*, *SPINK6*, *CHRM2*, *TG*, *CLLU1OS*, *POSTN*, and *NETO1*) were the top associated genes on 7 chromosomes. Among these prognostic genes, 9 genes (*TNFRSF4*, *ZAP70*, *ERMN*, *SPINK6*, *SLC6A18*, *CLLU1OS*, *POSTN*, *NETO1*, and *IGLV3-22*) were unfavorable prognostic genes, and 5 favorable prognostic genes (*CXCL5*, *CHRM2*, *TG*, *CTSG*, and *CEACAM7*).

**Table 2:** The prognostic genes of PRAD BCR and their corresponded causal effects.

**Gene**	**Chromosome**	**ACE (95% CI)**	***P*-ACE**	**MOR (95% CI)**	***P*-MOR**
*TNFRSF4*	1	0.092 (0.022, 0.163)	0.011	1.454 (1.089, 1.940)	0.011
*ZAP70*	2	0.116 (0.044, 0.188)	0.002	1.597 (1.191, 2.143)	0.002
*ERMN*	2	0.135 (0.05, 0.219)	0.002	1.728 (1.222, 2.444)	0.002
*CXCL5*	4	-0.142 (-0.226, -0.059)	0.001	0.562 (0.399, 0.792)	0.001
*SPINK6*	5	0.153 (0.047, 0.26)	0.005	1.865 (1.201, 2.897)	0.005
*SLC6A18*	5	0.165 (0.017, 0.313)	0.029	1.944 (1.056, 3.578)	0.033
*CHRM2*	7	-0.173 (-0.266, -0.079)	<0.001	0.496 (0.337, 0.731)	<0.001
*TG*	8	-0.127 (-0.243, -0.012)	0.031	0.598 (0.372, 0.960)	0.033
*CLLU1OS*	12	0.13 (0.053, 0.207)	0.001	1.689 (1.233, 2.312)	0.001
*POSTN*	13	0.083 (0.009, 0.156)	0.027	1.398 (1.037, 1.885)	0.028
*CTSG*	14	-0.133 (-0.229, -0.038)	0.006	0.584 (0.395, 0.863)	0.007
*NETO1*	18	0.137 (0.023, 0.252)	0.019	1.740 (1.090, 2.778)	0.020
*CEACAM7*	19	-0.165 (-0.289, -0.042)	0.009	0.511 (0.307, 0.851)	0.010
*IGLV3-22*	22	0.166 (0.004, 0.328)	0.044	1.956 (1.001, 3.823)	0.050

*CI: confidence interval.

**Figure 5:**
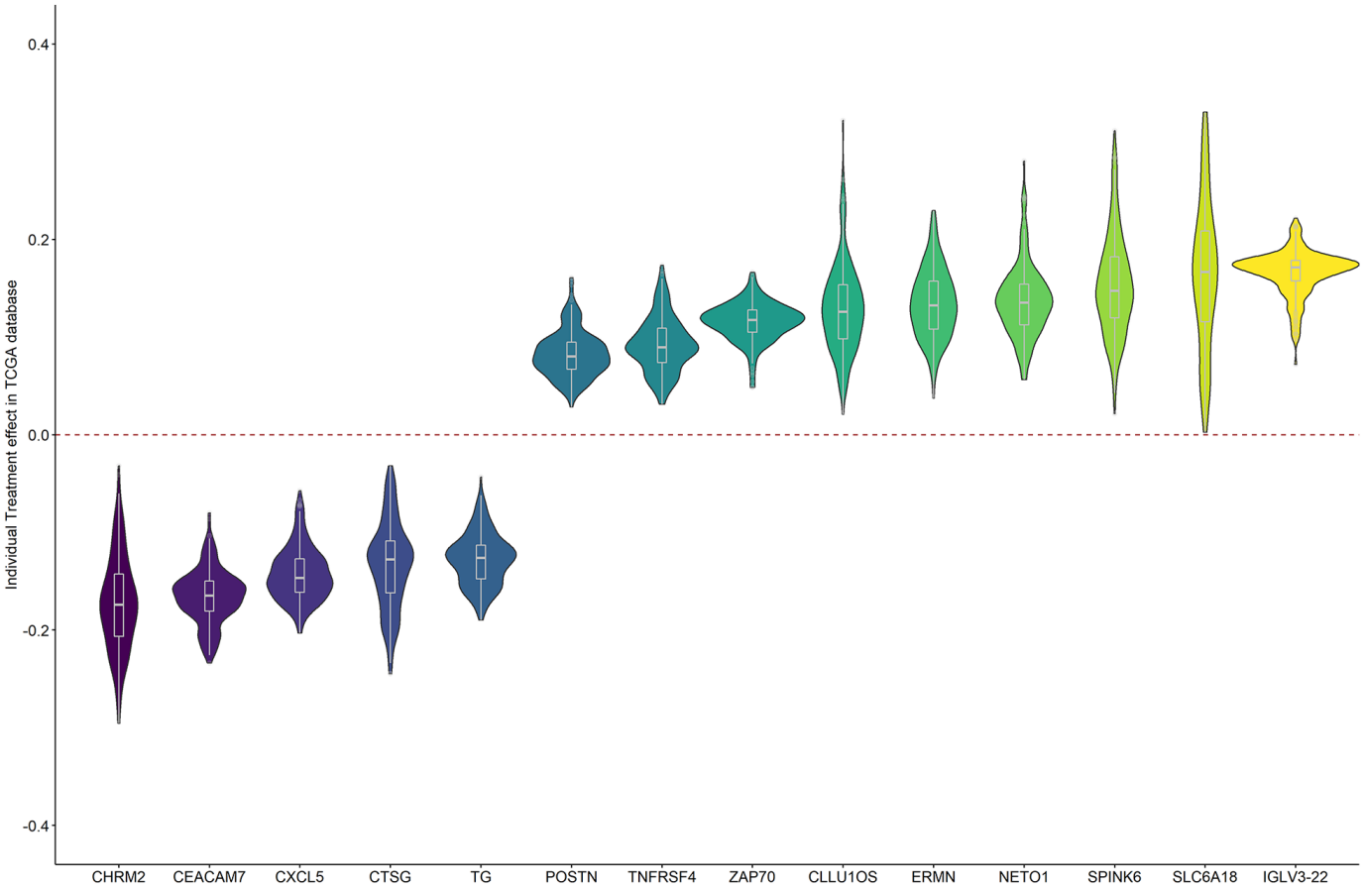
The individual causal effects of the 14 prognostic genes on PRAD BCR.

Figure 5 shows the individual treatment effects of each prognostic gene on BCR. The unfavorable genes were a risk for all patients, but with a range of positive causal effects. Similar results were obtained for the favorable genes.

### Functional analysis of the prognostic genes

**Figure 6:**
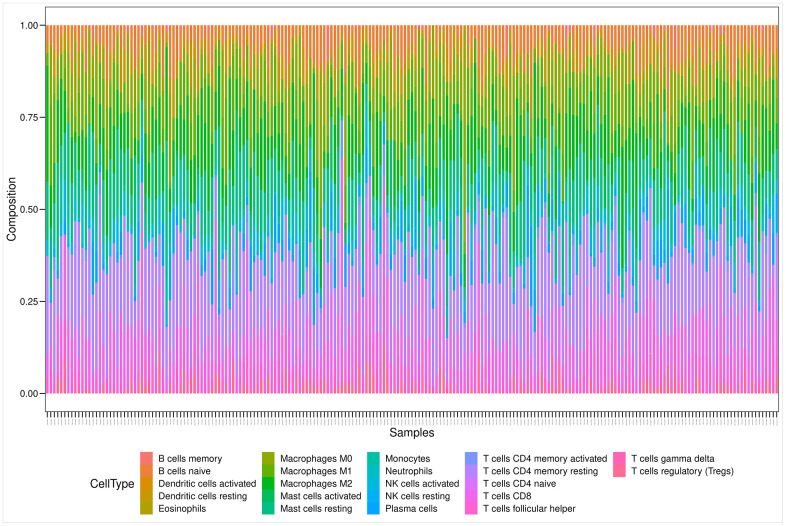
**Bar plot showing the proportion of 22 kinds of TIC profiles in PRAD tumor samples.** Rows and columns represent immune cell compositions and samples, respectively.

Figure 6 illustrates the estimated proportion of tumor-infiltrating immune components in the PRAD samples using the Cell-type Identification By Estimating Relative Subsets Of RNA Transcripts (CIBERSORT) analysis [25]. Among these immune cell profiles, CD4 memory resting T cells occupied the largest proportion in the PRAD samples. The Wilcoxon rank-sum test showed that only two types of tumor-infiltrating immune cells (plasma cells and follicular helper T cells) were not associated with the expression levels of the 14 prognostic genes.

Among the 14 identified prognostic genes, 6 genes (*TNFRSF4*, *ZAP70*, *CXCL5*, *CHRM2*, *CTSG*, and *IGLV3-22*) were reported to be associated with promoting antitumor immunity in previously published papers, 4 genes (*SPINK6*, *POSTN*, *CLLU1OS*, and *CEACAM7*) were associated with the metastasis of tumor cells, and 4 genes (*ERMN*, *SLC6A18*, *TG*, and *NETO1*) were associated with other diseases.

### Gene ontology term and Kyoto encyclopedia of genes and genomes pathway analysis of the prognostic genes

**Figure 7:**
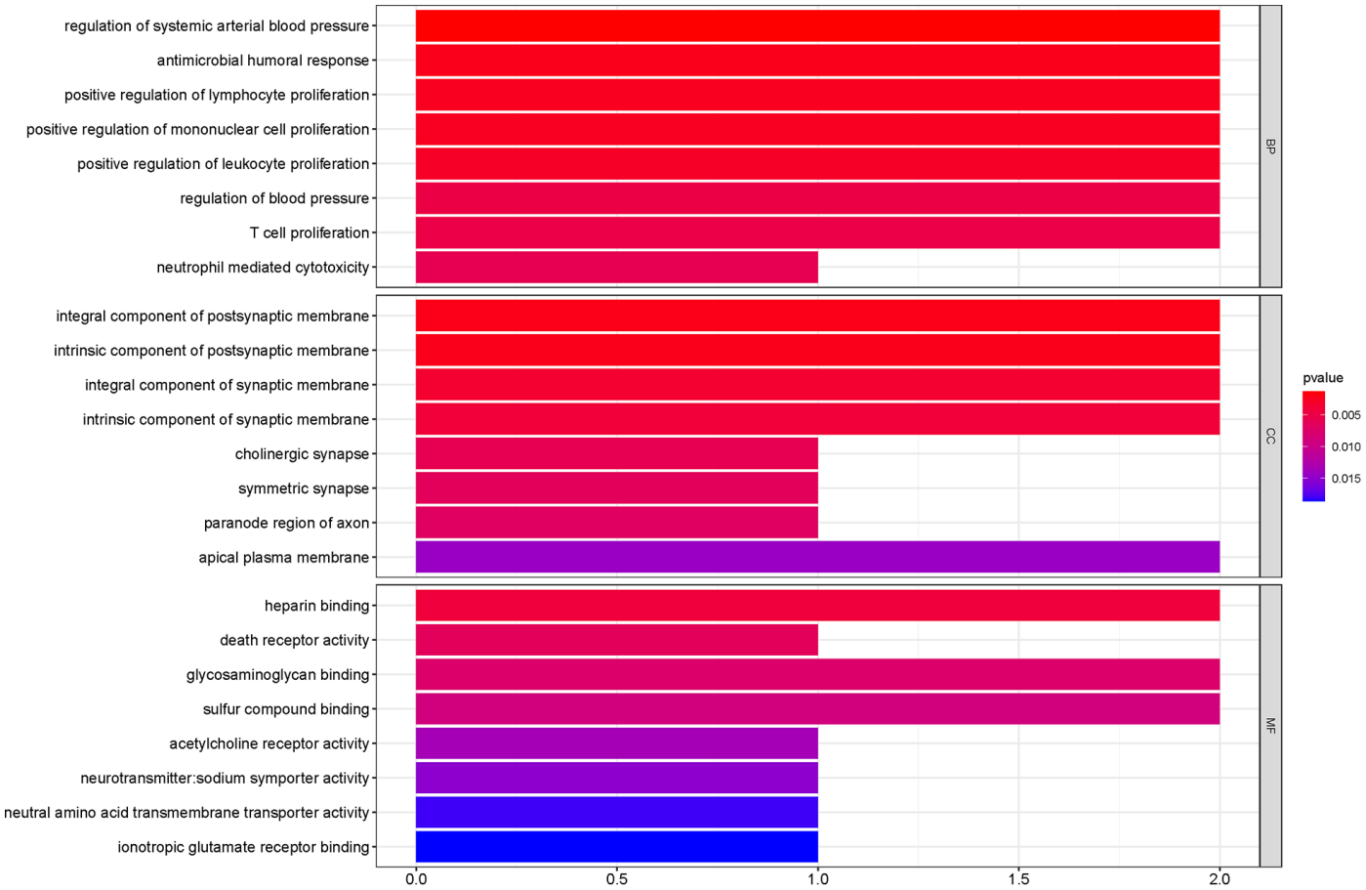
**GO term enrichment analyses of the prognostic genes.** The main GO terms (*P* values < 0.05) are shown for Biological process (BP), Cellular component (CC) and Molecular function (MF) respectively.

**Figure 8:**
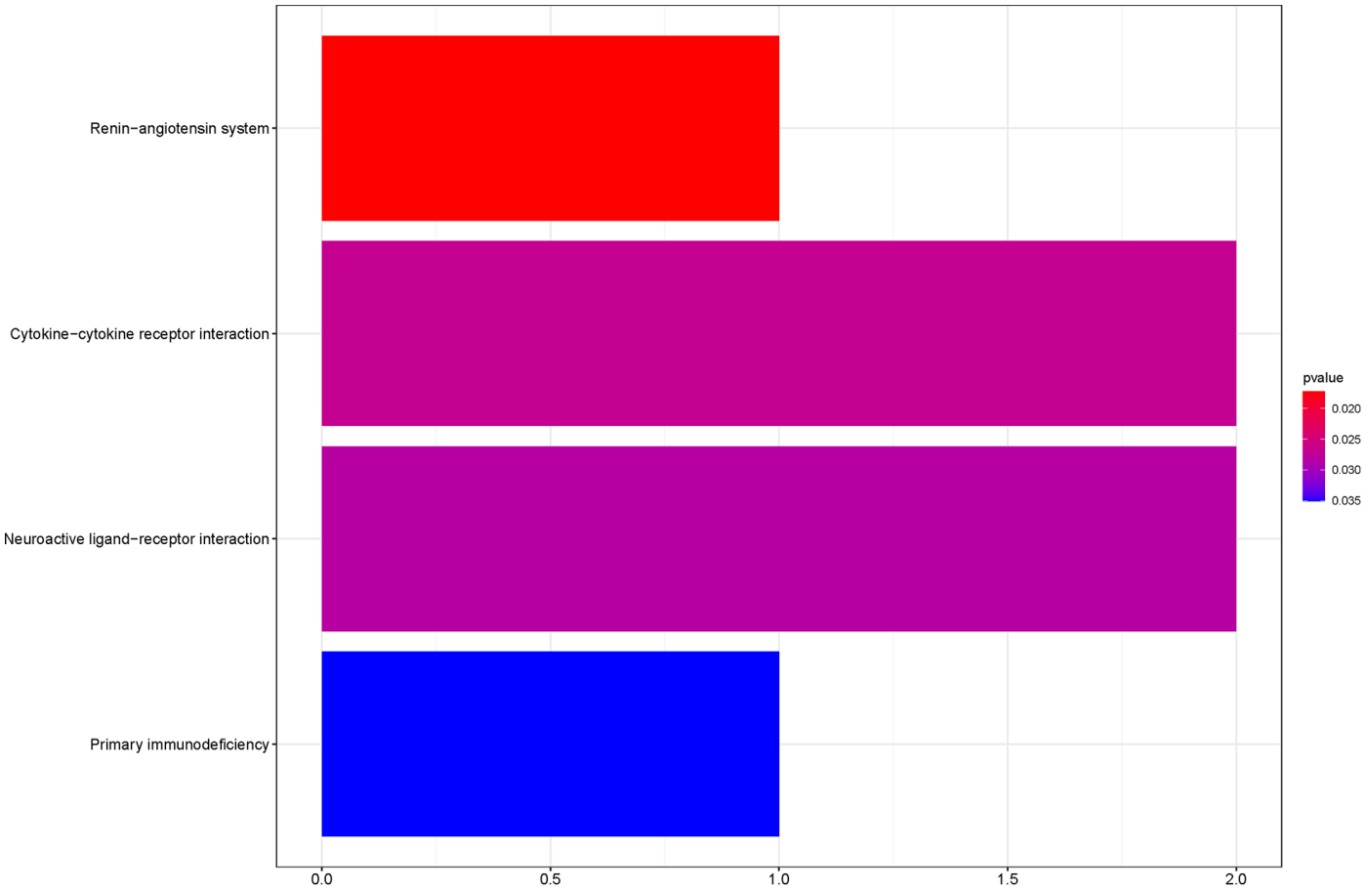
KEGG pathway analyses of the prognostic genes.

To outline the potential functions of the prognostic genes, we performed a functional enrichment analysis of the 14 prognostic genes. The results of gene ontology (GO) term enrichment analysis suggested strong correlations of these genes with immune responses (Figure 7). Kyoto encyclopedia of genes and genomes (KEGG) pathway analysis revealed the significant enrichment of four pathways. In particular, we detected that *ZAP70* was enriched in primary immunodeficiency, *TNFRSF4*, and *CXCL5* were enriched in cytokine-cytokine receptor interaction, *CTSG* was enriched in the renin-angiotensin system, and the *CHRM2* and *CTSG* were enriched in neuroactive ligand-receptor interaction (Figure 8). These results further illustrated that the two pathways derived from the KEGG analysis were associated with immune responses.

### Analysis of the correlations between prognostic genes and androgen receptor in stromal cells

**Figure 9:**
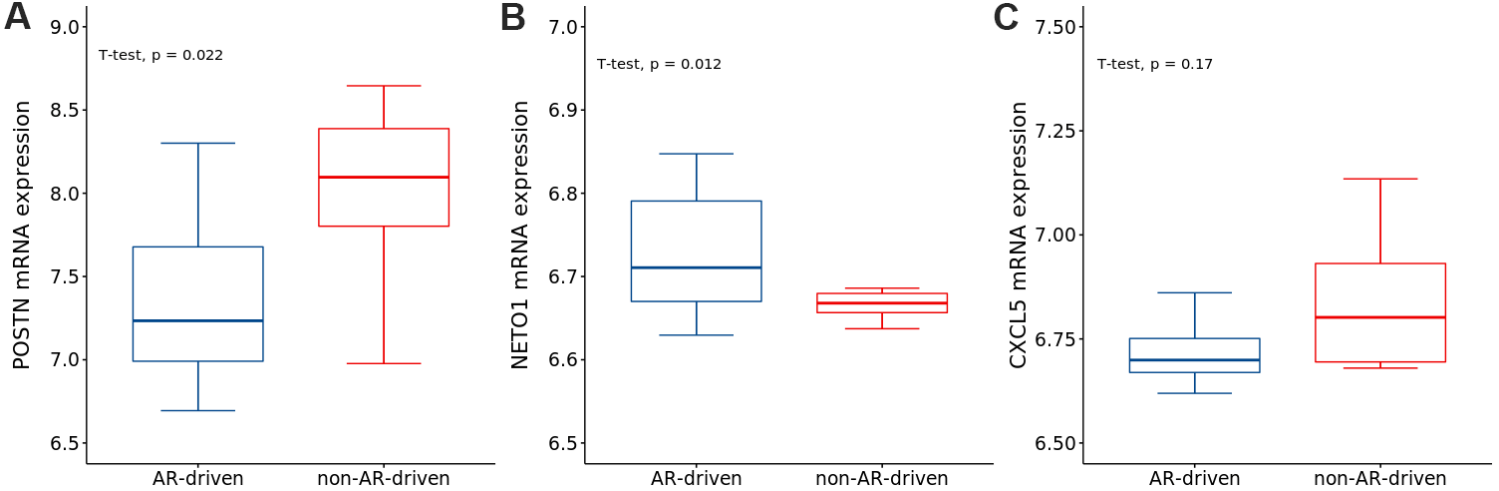
The expressions of (**A**) *POSTN*, (**B**) *NETO1* and (**C**) *CXCL5* in AR- and non-AR-driven groups using GSE101607. *T*-test was used to measure the difference between the two groups.

To explore the correlations between AR and the identified genes, we compared the expression of genes in the AR- and non-AR-driven groups using the GEO dataset (GSE101607). Our results showed that *POSTN* was significantly overexpressed in non-AR compared with AR-driven samples (Figure 9A), consistent with the findings of Cattrini et al. [26]. *NETO1* was overexpressed in AR-driven samples (Figure 9B). Although we did not detect any association between the expression of *CXCL5* and AR (Figure 9C), AR signaling has been reported to promote PRAD progression via modulation the AKT-NF-κB-*CXCL5* signaling [10].

### Validation in the GEO database

We further verified the 14 prognostic genes in an additional PRAD cohort obtained from the GEO database. We downloaded and analyzed the gene expression data of 203 PRAD cases in the GSE70770 dataset. A total of five genes (*ZAP70*, *CXCL5*, *SPINK6*, *CHRM2*, and *TG*) were significantly associated with early-onset BCR in PRAD (Table 3). The results of individual causal effects indicated that *CHRM2* and *SPINK6* might have different functions in different individuals with different features (Supplementary Figure 2).

**Table 3:** The validated genes in GEO database and their corresponded causal effects.

**Gene**	**Chromosome**	**ACE (95% CI)**	***P*-ACE**	**MOR (95% CI)**	***P*-MOR**
*ZAP70*	2	0.288 (0.226, 0.35)	<0.001	4.099 (2.895, 5.805)	<0.001
*CXCL5*	4	-0.14 (-0.256, -0.023)	0.018	0.425 (0.226, 0.798)	0.008
*SPINK6*	5	0.364 (0.294, 0.434)	<0.001	5.797 (3.881, 8.658)	<0.001
*CHRM2*	7	-0.211 (-0.271, -0.151)	<0.001	0.34 (0.247, 0.469)	<0.001
*TG*	8	-0.221 (-0.325, -0.116)	<0.001	0.199 (0.094, 0.423)	<0.001

## DISCUSSION

The aim of this study was to identify TME-related biomarkers, implicated in the development of BCR after prostatectomy, using the semi-parametric targeted approach, TMLE. TME is known to be comprised of a complex mixture of tumor-associated fibroblasts, infiltrating immune cells, endothelial cells, extracellular matrix proteins, and signaling molecules, such as cytokines [27–29]. Both immune and stromal cells have been proposed to be valuable for tumor diagnosis and prognosis evaluation. Similar to many other solid tumor types, prostate cancer is characterized by a rich tumor-stroma interaction network that forms the TME [27–29]. Our results also revealed that the TME (immune and stromal scores) was associated with early-onset BCR of patients with PRAD.

In addition, BCR serves as an indicator of the early stages of relapse, as local recurrence and distant metastasis might occur after BCR [30]. Furthermore, as BCR within 3 years of surgery is a critical node for prostate-specific mortality [3], it is necessary to identify the risk factors for 3-year BCR in the TME.

Using the traditional approaches, such as linear or logistic regression models, confounding factors and complex associations among covariates might bias the results and lead to fallacious conclusions. Whereas, robust TMLE was demonstrated to help reduce the risk of spurious findings [17]. Although TMLE optimizes the bias-variance tradeoff for the estimated causal effects, a rough trend could still be observed for the individual effects of patients. Based on this strategy, we identified 14 genes involved in the prognosis of PRAD.

*TNFRSF4* (also known as *OX40* or *CD134*) is a member of the tumor necrosis factor receptor superfamily, subserving co-stimulatory functions of T-cells during infection [31–33]. It is predominantly and transiently expressed by both human CD4+ and CD8+ T cells [32]. Studies have shown that regulatory T cells express more *TNFRSF4* than conventional CD4+ T cells in multiple human tumors [34]. Several anti-*TNFRSF4* agonistic monoclonal antibodies are currently being tested in early-phase cancer clinical trials [31]. The expression of *TNFRSF4* on tumor-infiltrating lymphocytes (TILs) has been studied in different tumor types, such as breast cancer, melanoma, B-cell lymphoma and head and neck cancers [35–40]. In colon cancer, the high expression of *TNFRSF4* in TILs, mesenteric lymph nodes, or invasive margin lymphoid aggregates was reported to correlate with better overall survival [40]. In our study, *TNFRSF4* was expressed at high levels in the BCR group, indicating that *TNFRSF4* could be a marker of BCR status in PRAD.

*ZAP70*, a 70 kDa tyrosine kinase of the Syk family, has been reported to significantly promote tumor angiogenesis and immunosuppression in cancer cell lines or samples [41–43]. Richardson et al. reported that *ZAP70* was activated in response to migratory and survival signals in B-cell chronic lymphocytic leukemia [44]. Fu et al. found that *ZAP70* was overexpressed in prostate cancer cell lines and tissues, facilitating prostate cancer cell migration and invasion [45]. MiR-631 was shown to target the 3′-UTR of *ZAP70* mRNA and inhibit the expression of *ZAP70*, thereby inhibiting prostate cancer cell migration and invasion [45]. Our results showed that *ZAP70* was mapped to the primary immunodeficiency pathway in KEGG and expressed at high levels in patients with BCR. Combined with previous studies, we speculated that *ZAP70* might not only be an important regulator of cancer metastasis but also useful in predicting the BCR in patients with prostate cancer.

*CXCL5* is a proangiogenic CXC-type chemokine known to act as an inflammatory mediator and a powerful attractant for granulocytic immune cells. It is secreted by both immune (neutrophils, monocytes, and macrophages) and nonimmune (epithelial, endothelial, and fibroblastic) cell types [46]. Wang et al. pointed out that CXCL5 was a cancer-secreted chemokine that attracted *CXCR2*-expressing myeloid-derived suppressor cells (MDSCs) and, correspondingly, pharmacological inhibition of CXCR2 impeded tumor progression [47]. Biological, molecular and pharmacological analyses established that a Yap1-mediated CXCL5-CXCR2 signaling axis recruits MDSCs into the TME [47]. Moreover, AR signaling was shown to promote the progression of PRAD via modulation of the AKT-NF-κB-*CXCL5* signaling. Our results showed that *CXCL5* was a prognostic gene, suggesting that the inflammatory mediator, *CXCL5*, might be a potentially protective prognostic factor for prostate cancer.

*CHRM2*, which mediates various cellular responses, has been demonstrated to be a significant marker of cognitive flexibility [48] and a potential therapeutic target for gastric cancer [49]. We found that *CHRM2* was a significant gene of the 3-year PRAD BCR. Validation using the GEO dataset confirmed our findings. We speculated that *CHRM2* might be a prognostic factor for prostate cancer.

*IGLV3-22*, which is a membrane-bound or secreted glycoprotein produced by B lymphocytes, belongs to the immunoglobulin lambda variable 3 (IGLV3) family. B lymphocytes are important cell types involved in the immune response of mammals [50]. Reports have shown that 40% of tumor-infiltrating lymphocytes in some patients with breast cancer were B cells [51, 52], suggesting the critical roles of these cells in modulating tumor responses [50]. Likewise, *IGLV3-21*, an important paralog of *IGLV3-22*, was confirmed to be a risk factor for chronic lymphocytic leukemia [53–55]. Our results showed that *IGLV3-22* was a causative gene of PRAD and indicated that patients with higher expression of *IGLV3-22* might have a worse prognosis.

*CTSG* is a serine protease of the chymotrypsin family that is stored in the primary (azurophil) granules of polymorphonuclear neutrophils [56]. Proteases are known to increase peripheral and central inflammation by regulating the chemotaxis of immune cells and the production of cytokines and chemokines. Previous studies have suggested *CTSG* as a potential marker of chronic pain after surgery [57], granulopoiesis or leukemogenesis [58]. In this study, we found that *CTSG* was a protective causative gene of BCR in patients with PRAD. Targeting and suppression of *CTSG* was shown to potentially inhibit the antitumor immunity.

*POSTN* is a 90-kDa extracellular matrix protein that interacts with multiple integrins to coordinate a variety of cellular processes, including epithelial-to-mesenchymal transition and cell migration [59, 60]. Stromal *POSTN* has been shown to participate in the regulation of cancer stem cell maintenance and expansion during metastatic colonization [61]. Researchers have reported that *POSTN* functions as a progression-associated and prognostic biomarker in glioma via the induction of invasive and proliferative phenotypes [62]. In our study, we found that *POSTN* was an unfavorable biomarker of PRAD BCR.

*CLLU1OS* is located on chromosome 12q22. The 12q21.33-12q22 region is dense with the expressed sequence tags derived from the germinal center B cells and CLL cells and is highly accessible for transcription in the B cells [63]. Accordingly, we detected a high expression of *CLLU1OS* in the BCR group, indicating that *CLLU1OS* was a risk factor for prostate cancer.

*SPINK6*, which is overexpressed in tumors and highly metastatic nasopharyngeal carcinoma cells, has been reported as an independent unfavorable prognostic factor [64]. It has been reported to act as a functional regulator of nasopharyngeal carcinoma metastasis via the *EGFR* signaling. Our results showed that *SPINK6* was a risk gene for 3-year BCR in patients with PRAD.

*CEACAM7* is a human cellular adhesion protein that belongs to the immunoglobulin superfamily. It has been reported to have low expression in colorectal cancers [65]. Our results showed low expression of *CEACAM7* in the BCR group, suggesting a putative role in the initiation and progression of prostate cancer.

*ERMN* is an essential gene involved in cytoskeletal rearrangements during myelinogenesis [66], and acts as a primary target of the disrupted folate metabolism [67]. *SLC6A18* is a specific transporter for neurotransmitters, amino acids, and osmolytes such as betaine, taurine, and creatine [68]. *TG* expresses the protein precursor of thyroid hormones, which are essential for the growth, development, and control of metabolism in vertebrates [69, 70]. *NETO1* has been found to be abnormally expressed in human carcinomas [71]. Higher expression of *NETO1* in epithelial ovarian cancer tissue samples has been reported to lead to worse overall survival and a higher probability of bowel metastases [71, 72]. The associations between these four genes and prostate cancer warrant further investigations.

The interaction between PRAD and TME might have serious effects on tumor evolution, further influencing tumor resistance, recurrence, and overall prognosis. Wang et al. provided a detailed description of the mechanism by which the activation of tumor-inherent genes altered TME [62]. The present study focused on the genetic characteristics of the TME, stimulating the development of PRAD. Our results might provide a basis for further studies on the role of TME in PRAD. However, the underlying mechanism remains unclear. Eventually, the putative role of these genes in the prognosis of prostate cancer would require further evaluation in future studies.

In summary, we used TCGA dataset to identify the potential prognostic genes in PRAD. Using the associations between the immune/stromal scores and the prognosis of PRAD, we revealed a set of genes related to the TME and the BCR of PRAD. These findings might facilitate the prognosis of PRAD. Based on our study, previously neglected genes could be used as biomarkers for PRAD. Finally, further studies of these genes could provide a more comprehensive understanding of the potential relationship between the prognosis of PRAD and the TME.

## MATERIALS AND METHODS

### Data collection

The transcriptome RNA sequence read count data of 550 patients with PRAD and their corresponding clinical profiles were obtained from TCGA (https://portal.gdc.cancer.gov). We collected data from patients with primary prostate tumors and defined the outcome by BCR-free survival time from surgery (≤3 as case vs. >3 years as control). Patients who died within 3 years after surgery without BCR were excluded. Clinical characteristics of the patients including data of age at initial pathologic diagnosis, pathological Gleason score, clinical TNM stage, and radiation therapy were also collected. TNM stage for each individual was classified according to the eighth edition of the American Joint Committee on Cancer TNM staging manual. Samples with missing clinical information were excluded from this study.

For validation, an additional PRAD cohort of 293 patients was obtained with the accession number GSE70770 from the GEO database (https://www.ncbi.nlm.nih.gov/gds). Gene expression data and clinical data were also downloaded. The same exclusion criteria as those used in TCGA were applied to this cohort.

### Construction of tumor microenvironment

The immune and stromal components in TME were calculated based on the ESTIMATE algorithm [24] using the R package estimate. Immune, stromal, and ESTIMATE scores, corresponding to the levels of immune cells, stromal cells, and the sum of both, respectively, were obtained. These three scores were applied to assess the infiltration level of immune and stromal cells and tumor purity in tumor tissues.

### Identification of differentially expressed genes based on immune scores and stromal scores

All patients were classified into two groups based on the mediation of immune and stromal scores to explore the correlation between gene expression profiles and immune or stromal scores. We used the R package *DESeq2* to perform differentiation analysis of gene expression, and DEGs were generated by comparing the high and low score groups. DEGs with log2|fold change| > 1 and adjusted *P* value < 0.05, after Benjamini–Hochberg false discovery rate [73], were considered significant.

### Statistical analysis of associated genes and clinical covariates

The univariate logistic regression models were used to determine the DEGs to be considered as candidate causative genes of BCR involved in the immune and stromal cells. According to its median expression level, each gene in tumor samples was grouped into high- or low-expression groups. Significant clinical covariates for BCR status, which were selected as candidate covariates in subsequent analysis, were examined using *t*-tests (age at initial pathologic diagnosis and weight) and χ^2^tests (radiation therapy of patients, pathological Gleason score and clinical TNM stage).

### Selection of the minimal sets of confounding covariates

To select the minimal sets of confounding covariates between candidate causative genes and the BCR status, the R package *CovSel* was used to screen all candidate causative genes and clinical covariates. *G* = {*g_1_*, *g_2_*, …, *g_n_*} denoted the binary candidate causative genes, *X* = {*x_1_*, *x_2_*, …, *x_m_*} the selected clinical covariates, and *Y* the outcome 3-year BCR in patients with PRAD. In addition, {(*G*\*g_i_*) + *X*} denoted the complete covariate vector of the target gene *g_i_* and *Y*. We assumed that *W_G_* was the subset of confounding covariates {(*G*\*g_i_*) + *X*}, which satisfied Y ┴{(*G*\*g_i_*) + *X*}*\W_G_|W_G_*. *V_G_* was the minimal set of confounding covariates *W_G_* satisfying *G_┴_W_G_\V_G_|V_G_*, and the final confounding set of the target gene *g_i_* and *Y*.

### Estimation of the causal effects of genes on biochemical recurrence

Causal effects are commonly defined in potential outcomes [74], that is, the average causal effect (ACE), defined as E[*Y*(1) − *Y*(0)], the marginal odds ratio (MOR), defined as {E[*Y*(1)] × E[1 − *Y*(0)]} /{E[1 − *Y*(1)] × E[*Y*(0)]}, and the individual causal effect (ICE), defined as *Y*(1) − *Y*(0), where *Y*(1) is the outcome under exposure (*G* =1) and *Y*(0) is the outcome when unexposed (*G* = 0).

To causally interpret the causal effects, we put forward the following assumptions. i) Stable-unit-treatment-value assumption: the potential outcomes of a given individual will not be affected by their exposure status. ii) No unmeasured confounders: all the covariates altering the exposure and outcome are measured, formulated as (*Y*(1), *Y*(0))^┴^*G*|*V_G_*. iii) Positivity: every individual has a non-zero probability conditioning on the covariates within strata of *G*, which could be formulated as 0 < P(*G* = 1|*V_G_*) < 1. Thus, the ACE could be defined as E*_V_* [E (*Y* |*G* = 1, *V_G_*) − E (*Y* |*G* = 0, *V_G_*)] with the observed dataset.

In this study, TMLE was used to detect the causative genes and estimate the causal effects, including ACE, MOR, and ICE. TMLE used two steps to target the optimal bias-variance tradeoff and obtain the target parameters. First, we conditionally estimated the expectation of the outcome with both exposures and confounders, E(*Y* | *G*, *V_G_*), which was used to predict the potential outcomes. Second, we evaluated the exposure mechanism P(*G* = 1| *V_G_*) to update the estimation of E(*Y* | *G*, *V_G_*). We eventually used the final updated estimation of E(*Y* | *G*, *V_G_*) to predict a pair of potential outcomes for each individual, and calculate both ACE and ICE. The ICE was calculated as the difference between these pairs of each individual, whereas ACE was the average difference of ICE. Then, we calculated MOR as the ratio of the two odds of BCR occurring in the high- and low-expression groups. Combining TMLE with Super Learner, we selected five models (logistic regression model with or without interaction, elastic net regression, BART, and random forest, using the R functions *glm*, *glm interaction*, *glmnet*, *bartMachine*, and *randomforest*, respectively) to build both outcome and propensity score models to improve the robustness and precision of our estimates.

### Functional annotation and analysis

To elucidate the biological functions of the prognostic genes with the immune microenvironment, we performed CIBERSORT analyses to estimate the proportion of tumor-infiltrating immune components in PRAD samples. The Wilcoxon rank-sum test was used to determine the association between the expression level of the prognostic genes and 22 types of immune cell profiles. Statistical significance was set at *P* < 0.05.

We performed GO and KEGG pathway enrichment analyses to investigate the shared biological functions among the identified genes that were common among the high and low immune/stromal score groups. The enrichment analyses were performed using the R packages *clusterProfiler*, *enrichplot*, and *ggplot2*. Only terms with adjusted *P* < 0.05 by FDR were considered as significantly enriched. To explore the correlations between AR and the identified genes, we compared the expression of genes in AR- and non-AR-driven groups using the GEO dataset (GSE101607).

All statistical analyses were performed using the software R 3.6.2 from CRAN (http://cran.r-project.org/). *P* values were corrected using the Benjamini–Hochberg method to control the FDR for multiple testing when appropriate [73].

## Supplementary Material

Supplementary Figures
