# Supplementary material for: Identification of microenvironment related potential biomarkers of biochemical recurrence at 3 years after prostatectomy in prostate adenocarcinoma
Source: Aging (Albany NY). 2021 Jun 16;13(12):16024–42. doi: 10.18632/aging.203121 (PMC8266350; doi:10.18632/aging.203121)
Supplement: Supplementary Figures [file aging-13-203121-s001.pdf]

SUPPLEMENTARY FIGURES

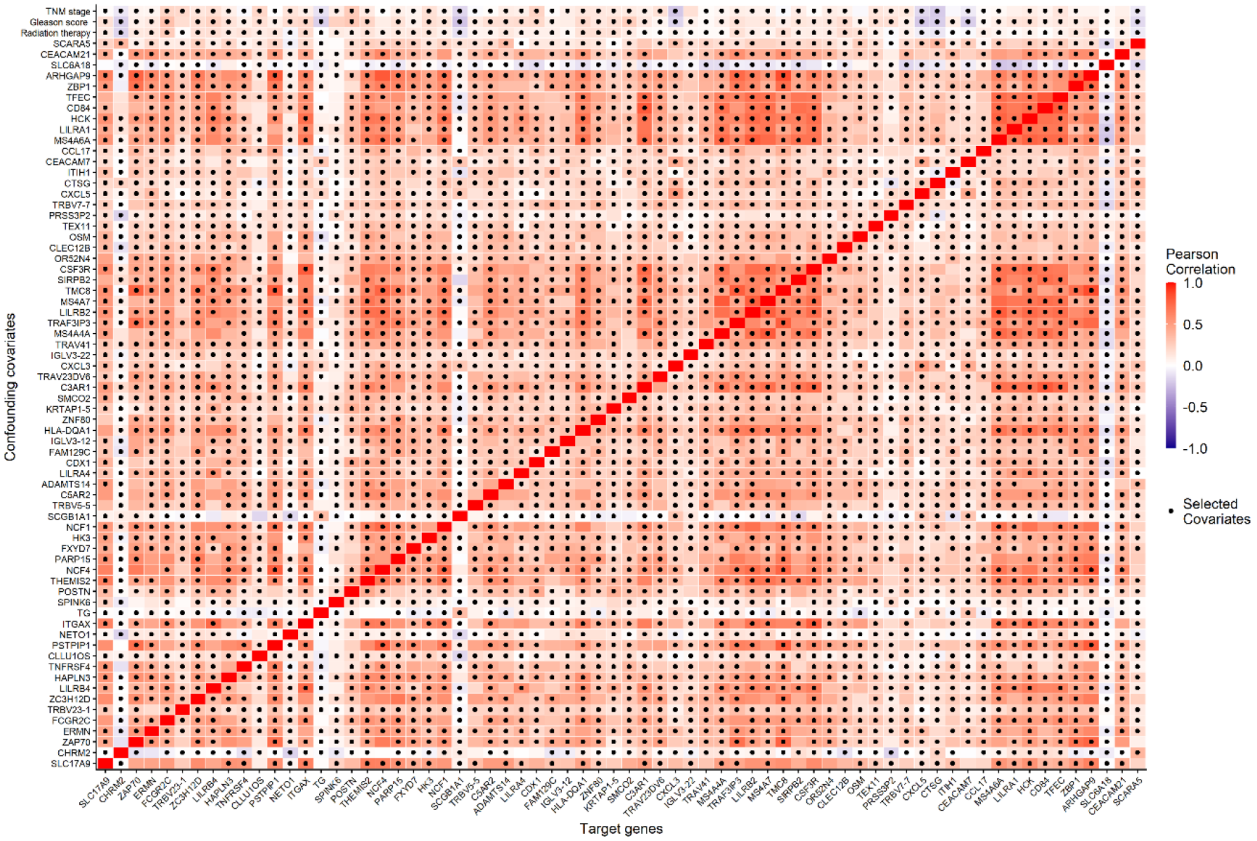

Supplementary Figure 1. The Pearson correlation coefficients (the corresponding color) of the 68 candidate genes (horizontal axis) and the 70 candidate confounding covariates (vertical axis), as well as the covariate set W_G that the candidate confounding covariates after removing variables W_G would be conditionally independent of outcome Y given W_G (the dark dot of each column).

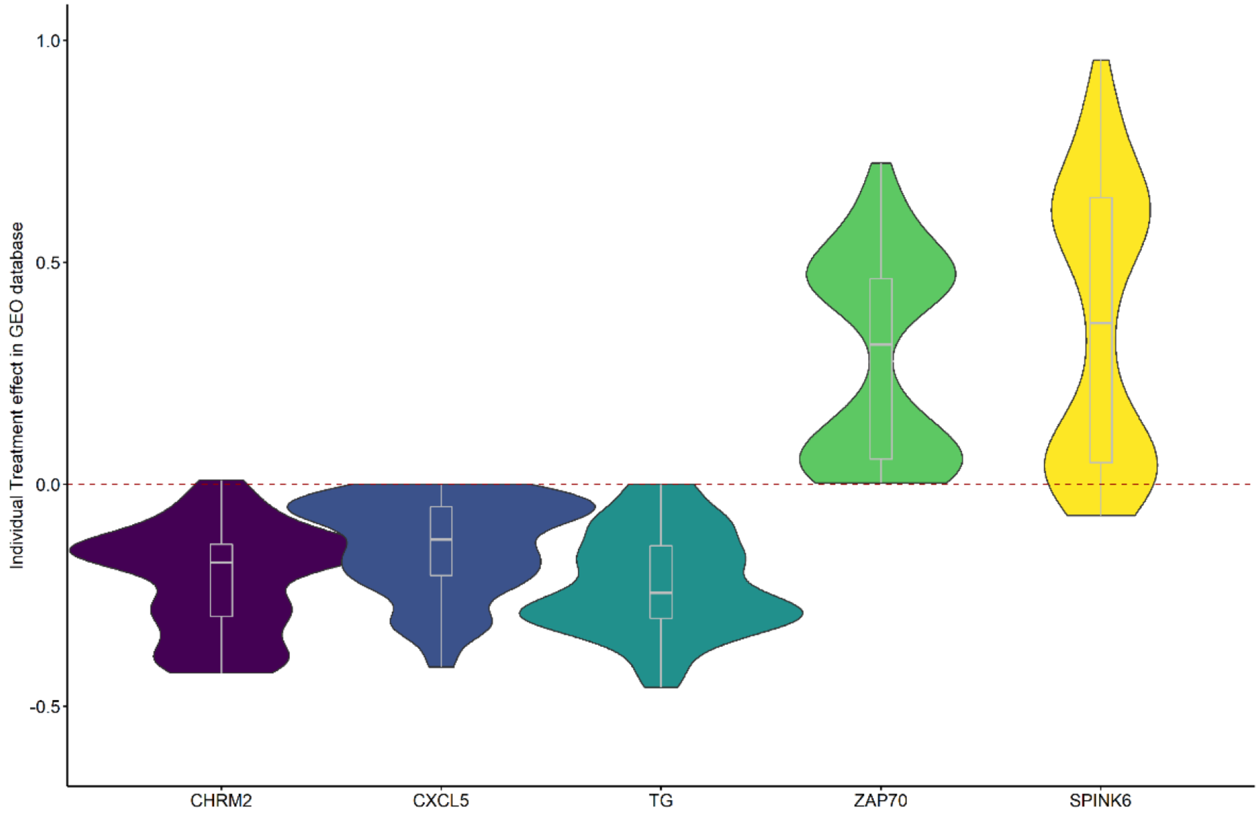

Supplementary Figure 2. The individual causal effects of the validated genes in the GEO dataset.
